# Experimental and Statistical Analyses of Dimensional Accuracy of Geometry-Dependent Features in Fiber Laser Cutting of CFRP

**DOI:** 10.3390/ma19204352

**Published:** 2026-10-16

**Authors:** Viliam Čačko, Iveta Čačková, Bálint Ferenczi, Shane Shabu, Alena Brusilová, Zuzana Gábrišová, Ľubomír Šooš

**Affiliations:** Institute of Production Engineering and Quality, Faculty of Mechanical Engineering, Slovak University of Technology in Bratislava, Nám. slobody 17, 812 31 Bratislava, Slovakia; viliam.cacko@stuba.sk (V.Č.); iveta.cackova@stuba.sk (I.Č.); balint.ferenczi@stuba.sk (B.F.); alena.brusilova@stuba.sk (A.B.); zuzana.gabrisova@stuba.sk (Z.G.); lubomir.soos@stuba.sk (Ľ.Š.)

**Keywords:** CFRP, fiber laser cutting, dimensional accuracy, laser power, cutting speed, operating mode, design of experiments, regression modeling

## Abstract

This study investigates the influence of fiber laser cutting parameters on the dimensional accuracy of carbon fiber-reinforced polymer (CFRP) components. A full factorial design of experiments was employed considering laser power, cutting speed, and laser operating mode as input factors. Three laser power levels (800, 1600, and 2000 W), three cutting speeds (250, 500, and 1000 mm/min), and two operating modes (continuous wave, CW, and gated pulse, GP) were evaluated. Dimensional accuracy of 90 laser-cut specimens was assessed using three selected geometrical responses—the inner width (*L*_2_) and two internal feature heights (*a*_1_ and *a*_3_)—followed by analysis of variance and regression modeling. Laser power was identified as the dominant factor for *L*_2_ and *a*_1_, whereas *a*_3_ exhibited pronounced nonlinear behavior governed primarily by cutting speed and its quadratic contribution. The operating mode significantly affected *L*_2_ and *a*_3_, while its influence on *a*_1_ was statistically insignificant. The pronounced nonlinear behavior of *a*_3_ may be associated with the combined effects of heat accumulation, anisotropic heat transfer through the carbon-fiber reinforcement, and thermally induced degradation and recession of the polymer matrix. Differences in temporal energy delivery between CW and GP operation may influence the local thermal conditions and consequently affect material removal and the resulting position of the cut edge. The developed regression models achieved *R*^2^ values of 90.37%, 87.25%, and 92.22% for *L*_2_, *a*_1_, and *a*_3_, respectively. The calculated nominal line energy ranged from 48 to 480 J/mm and supported the observed tendency that higher power combined with lower cutting speed was generally associated with increased dimensional deviation. The results demonstrate that dimensional accuracy in fiber laser cutting of CFRP is strongly geometry-dependent and requires feature-specific process optimization rather than a single universal cutting condition.

## 1. Introduction

Laser beam machining (LBM) represents an important non-conventional manufacturing technology widely applied to advanced engineering materials due to its high precision, flexibility, and contactless nature. In contrast to conventional machining, laser cutting eliminates direct mechanical interaction between the cutting tool and workpiece, thereby avoiding cutting forces and physical tool wear. These characteristics are particularly advantageous when processing heterogeneous and difficult-to-machine materials, for which conventional cutting methods may lead to excessive tool wear, mechanical damage, or reduced dimensional stability [1,2].

Polymeric materials and polymer–matrix composites are being increasingly employed in structural components, transportation, energy systems, coatings, electronics, and biomedical applications because of their low density, corrosion resistance, processability, and highly tailorable physical and mechanical properties. Their broader engineering application is nevertheless limited by temperature sensitivity, creep, fatigue, abrasion, microcracking, and, in some structural applications, lower stiffness and load-bearing capability compared with metallic systems. Reinforcement with fibers or particulate fillers can significantly improve mechanical performance; however, the resulting properties depend strongly on filler content, dispersion, interfacial bonding, and defect formation. Excessive filler loading may promote agglomeration and void formation and consequently reduce mechanical performance, while advanced approaches such as self-healing polymer nanocomposites are being developed to improve damage tolerance and service life [3,4].

Carbon-fiber-reinforced polymers (CFRPs) are widely used in aerospace, automotive, energy and other high-performance engineering applications due to their high specific strength and stiffness, low density, corrosion resistance and favorable fatigue behavior [1,2]. Despite these advantages, CFRP materials remain difficult to machine because of their heterogeneous and anisotropic structure. Conventional milling, drilling, and trimming may result in rapid tool wear, delamination, fiber pull-out, matrix cracking, and edge damage. The resulting quality is additionally influenced by fiber orientation and the mechanical response of the individual constituent phases [1,2]. Laser cutting therefore represents an attractive alternative for producing complex CFRP geometries without mechanical loading of the workpiece.

The quality of laser-cut CFRP components is strongly influenced by the applied processing conditions. Laser power and cutting speed directly affect the amount of energy delivered to the material, while the operating regime determines the temporal characteristics of the energy input. Previous studies have demonstrated that inappropriate processing conditions may lead to non-uniform material removal, matrix recession, exposed fibers, thermal damage, and deterioration of the resulting cut geometry [5,6,7,8]. Leone et al. [5] investigated fiber laser cutting of thin CFRP sheets and demonstrated the significant influence of processing parameters on cut quality. Li et al. [6] used a factorial experimental approach to investigate fiber laser machining of woven CFRP laminates, while Oh et al. [7] showed that laser power, scanning speed, and the number of passes strongly affect the resulting cut characteristics. The influence of continuous and pulsed energy delivery has also been investigated, demonstrating that the laser operating regime can significantly modify the interaction between the composite material and laser beam [8].

Laser–material interaction in CFRP is further complicated by the pronounced thermal and structural heterogeneity of the laminate. Carbon fibers exhibit substantially higher thermal conductivity along the fiber direction than transverse to it, resulting in anisotropic heat propagation and a dependence of the local thermal field on the orientation of the cutting path relative to the reinforcement. Experimental investigations have demonstrated that fiber orientation and laminate lay-up can significantly affect kerf geometry, heat-affected zone(HAZ) development, matrix recession, fiber exposure, and delamination. The polymer matrix is particularly sensitive to thermal degradation because its decomposition temperature and energy required for removal are substantially lower than those of the carbon fibers. Consequently, preferential matrix removal can occur before complete fiber ablation, producing local edge recession and geometrical distortion [9,10,11].

The type of polymer matrix also affects the thermal response during laser processing. Thermoset matrices such as epoxy undergo irreversible thermal decomposition, whereas thermoplastic matrices such as polyether ether ketone (PEEK) exhibit different softening and degradation kinetics and therefore require different thermal processing windows. The matrix system must consequently be considered when transferring optimized laser parameters between different CFRP materials [12].

Statistical methods provide an effective approach for evaluating the simultaneous influence of multiple laser-processing parameters. Design of experiments (DOE), analysis of variance (ANOVA), regression modeling and response surface methodology have been successfully applied to identify significant factors, quantify interaction effects and develop predictive models for laser cutting processes. Rao et al. [13] applied response surface methodology to fiber laser cutting of CFRP and demonstrated the applicability of statistical modeling for process optimization. Similar methodologies have also been successfully employed for evaluating dimensional accuracy in fiber laser cutting of metallic materials, where the resulting dimensional deviations were shown to depend on both individual process parameters and their interactions [14,15,16].

Despite these advances, most experimental studies continue to evaluate local cut-quality indicators such as kerf width, HAZ, taper, and edge morphology, while considerably less attention has been devoted to dimensional deviations of multiple geometrical features within complex computer-aided design (CAD)-defined contours.

However, most previous investigations of laser processing of CFRP have focused primarily on thermal damage, kerf characteristics, surface morphology, taper, or material-removal behavior [5,6,7,8,13]. Comparatively less attention has been devoted to the dimensional and geometrical accuracy of complete CFRP components containing several different CAD-defined features. This aspect is important because acceptable surface quality or limited thermal damage does not necessarily imply that the manufactured component accurately reproduces its nominal geometry. In practical manufacturing applications, dimensional conformity of the final component is therefore a critical quality requirement in addition to conventional cut-quality indicators.

In contrast to studies primarily focused on thermal and kerf-related characteristics, the present work provides a systematic evaluation of the dimensional and geometrical accuracy of CFRP components produced by fiber laser cutting. The combined influence of laser power, cutting speed, and laser operating mode is investigated using a mixed-level factorial experimental design. Multiple linear, radial, and angular geometrical characteristics of the manufactured specimens are evaluated using automated optical measurement, allowing the sensitivity of different geometrical features to the selected processing conditions to be compared.

Thus, the primary aim of this study is to experimentally investigate the influence of laser power, cutting speed and laser working mode on the dimensional accuracy of CFRP components cut by fiber laser. Statistical analysis is used to identify significant process factors and interactions and to develop predictive regression models for the evaluated dimensional responses. Based on previous observations of laser–material interaction, it is hypothesized that laser power and cutting speed have the dominant influence on dimensional deviations, whereas the influence of the laser operating mode is expected to be dependent on the evaluated geometrical characteristic. The results are intended to provide a quantitative basis for selecting processing conditions that improve the dimensional accuracy of CFRP components within the investigated experimental domain.

The specific contribution of the present study is the geometry-resolved evaluation of dimensional accuracy across multiple CAD-defined features of a single CFRP component under a statistically controlled 3 × 3 × 2 full factorial design. In contrast to studies focused primarily on HAZ, kerf width, or surface morphology, the proposed approach directly links laser power, cutting speed, and operating mode to feature-specific dimensional deviations and predictive regression models. The results therefore provide an engineering-oriented basis for selecting laser-processing conditions according to the critical geometrical feature of the component rather than relying on a single global cut-quality indicator.

## 2. Materials and Methods

### 2.1. Materials

The experiment was carried out on commercially available carbon fiber-reinforced polymer (CFRP) laminate sheets (KAVAN, Pardubice, Czech Republic, product code KAV60.3801) with nominal plate dimensions of 400 mm × 250 mm and a thickness of 1 mm. According to the supplier specifications, the laminate was manufactured using 3K carbon-fiber reinforcement arranged in a twill weave and had a matte surface finish. The nominal mass of each plate was 185 g, corresponding to an estimated bulk density of approximately 1.85 g/cm^3^. Detailed information concerning the polymer matrix composition, fiber volume fraction, individual ply thickness, and complete stacking sequence was not provided by the supplier.

CFRP materials combine high specific strength and stiffness with low density, corrosion resistance, and dimensional stability, which has resulted in their increasing application in aerospace, automotive, and other high-performance engineering sectors [1,2]. Nevertheless, their heterogeneous and anisotropic structure presents considerable challenges during secondary machining and edge-finishing operations [1,2].

Laser processing represents a promising alternative to conventional mechanical machining of CFRP because it provides contactless material removal and eliminates cutting-tool wear. However, the substantially different thermal and optical properties of the carbon fibers and polymer matrix result in complex material-removal mechanisms and may generate matrix recession, thermal degradation, heat-affected regions, and dimensional deviations. Previous experimental investigations of fiber laser cutting of CFRP have demonstrated that laser power and cutting speed substantially affect the resulting cut geometry and thermally induced damage [5,6,7,8]. 

The material thickness was maintained constant at 1 mm throughout the experimental campaign to eliminate thickness-related variability and enable a direct assessment of the influence of the investigated process parameters.

### 2.2. Fiber Laser Cutting System

**Figure 1:**
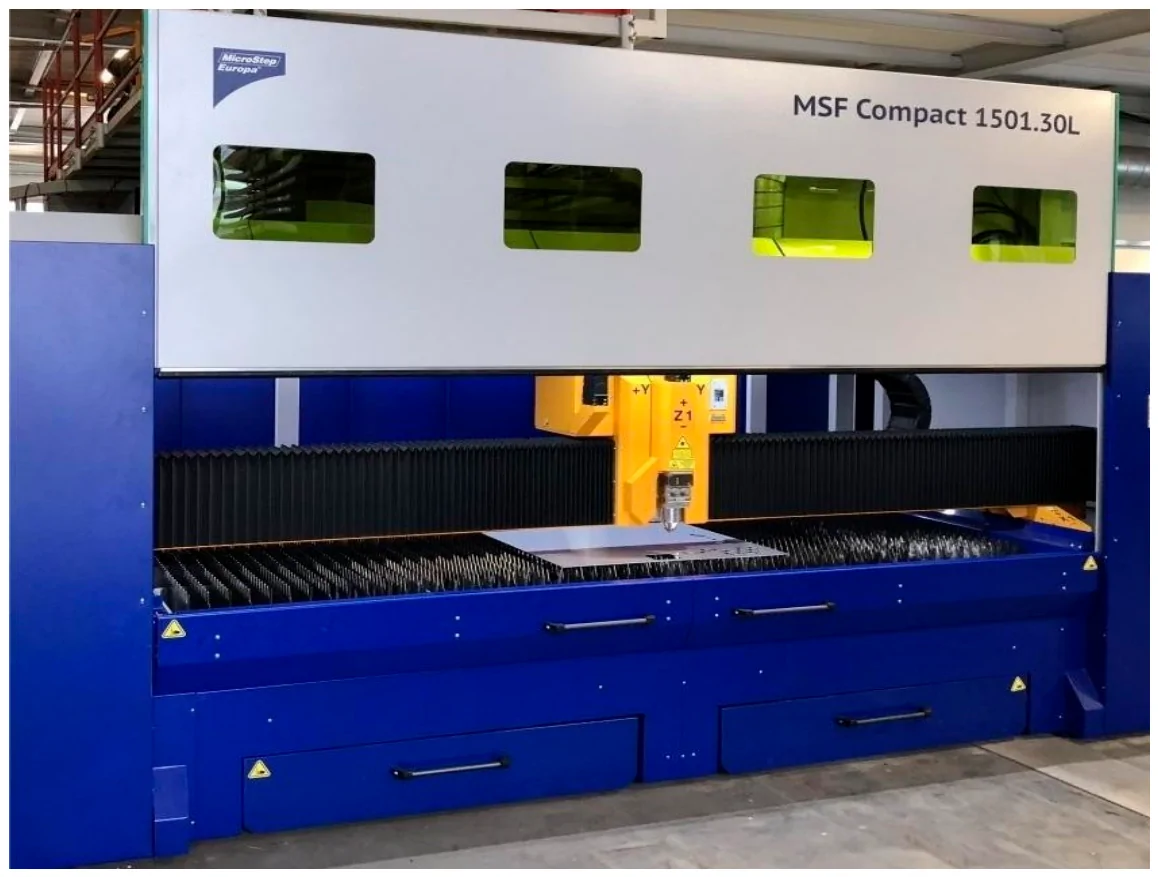
Fiber laser cutting machine used for the experimental investigation: MicroStep MSF 1501.30L.

The cutting experiments were performed on the MicroStep MSF 1501.30L (Figure 1) Computer numerical control(CNC) fiber laser cutting machine (MicroStep, Bratislava, Slovakia) with an IPG YLR-4000 fiber laser (IPG Photonics Corporation, Oxford, MA, USA) source. The machine provides an effective working area of 1.5 m × 3 m and a positioning repeatability of 0.03 mm. The maximum translational velocity of the individual X and Y axes is 130 m/min, while the maximum acceleration applicable during cutting is 2000 mm/s^2^ [17]. The same class of industrial CNC fiber laser systems has previously been successfully employed for systematic investigations of dimensional accuracy and process optimization in laser cutting [15,16].

Nitrogen (Linde, Tuhovská 3, 831 06 Bratislava, Slovakia) was employed as the assist gas throughout the experimental campaign at a constant pressure of 0.5 bar. As an inert gas, nitrogen was selected to suppress oxidation and the associated exothermic contribution during laser processing. The gas flow also assists in removing vaporized or thermally degraded matrix products and loose carbonaceous debris from the kerf while providing a local cooling effect. Maintaining a constant assist-gas pressure ensured that gas-related effects did not interfere with the evaluation of laser power, cutting speed, and operating mode. A nozzle with an outlet diameter of 1.5 mm was used. The focal position was maintained at the upper surface of the CFRP laminate (0 mm), while the nominal stand-off distance between the cutting head and the workpiece surface was 2 mm. The focal lengths of the collimating and focusing lenses were 100 mm and 175 mm, respectively. Keeping these parameters constant throughout the experiments isolated the effects of the selected independent variables.

Two laser operating regimes were investigated: continuous-wave (CW) and gated pulsed (GP) operation. For the pulsed regime, a pulse frequency of 5000 Hz and a duty cycle of 99% were employed. Owing to the limited flatness of the CFRP laminates and supporting cutting table, the actual focal and stand-off positions were estimated to vary within approximately ±0.2 mm. No kerf compensation was applied in the cutting program. The laser beam followed the nominal CAD contour. 

Prior to contour cutting, a piercing operation was performed using a piercing time of 0.1 s and a laser power of 3000 W. For pulsed-mode piercing, an assist-gas pressure of 0.5 bar, pulse frequency of 1000 Hz, duty cycle of 30%, and focal position of −1 mm relative to the upper laminate surface were employed. 

### 2.3. Experiment

The systematic effect of the chosen laser processing parameters on the dimensional accuracy of the CFRP specimens was investigated by a full factorial design of experiments (DOE). Factorial experimental designs provide an effective framework for identifying both individual factor effects and interactions between process variables and have been successfully applied to the optimization of fiber laser cutting processes [13,15,16]. Similar statistical approaches have also been employed in previous investigations of dimensional accuracy in laser-cut metallic materials [14,15,16].

Three independent process parameters were considered: cutting speed *v*, laser power *P*, and laser operating mode *M*. Cutting speed and laser power were treated as quantitative factors, whereas the operating mode was considered a categorical factor.

Cutting speed and laser power were each investigated at three levels, and two laser operating modes were evaluated. The factor levels were determined on the basis of preliminary cutting trials performed on the same CFRP laminate prior to the main experimental campaign. These trials were used to identify the processing window within which complete through-cutting of the 1 mm thick material was consistently achieved while avoiding excessive thermal damage of the cut edges. The laser power levels (800, 1600, and 2000 W) and cutting speeds (250, 500, and 1000 mm/min) were selected to span this window, from conditions of comparatively low to high nominal energy input. Continuous-wave (CW) and gated-pulsed (GP) operation were included, as these represent the standard operating regimes available on the industrial cutting system used. The combination of three cutting-speed levels, three laser-power levels, and two operating modes resulted in a 3 × 3 × 2 full factorial design comprising 18 unique experimental conditions. Each condition was replicated five times, resulting in a total of 90 independently produced specimens.

To minimize systematic effects associated with cutting sequence, gradual optical contamination, and possible temporal drift of the laser system, the experimental runs were randomized. The five replications were treated as five independent experimental blocks. Within each block, all 18 parameter combinations were performed once in a randomized order generated prior to the experiment. A different random sequence was used for each replication block. This procedure distributed potential time-dependent changes in the laser system across the complete experimental domain and reduced the likelihood that such effects would be confounded with a particular laser power, cutting speed, or operating mode. 

The selected factor levels are shown in Table 1.

### 2.4. Specimen Geometry and Evaluated Dimensional Characteristics

A dedicated test specimen containing multiple linear, radial, and angular geometrical features was used to evaluate the dimensional accuracy achieved during fiber laser cutting of CFRP. The specimen geometry was designed to provide several distinct features within a single cutting contour, enabling the influence of the investigated process parameters to be assessed across different regions of the component.

**Figure 2:**
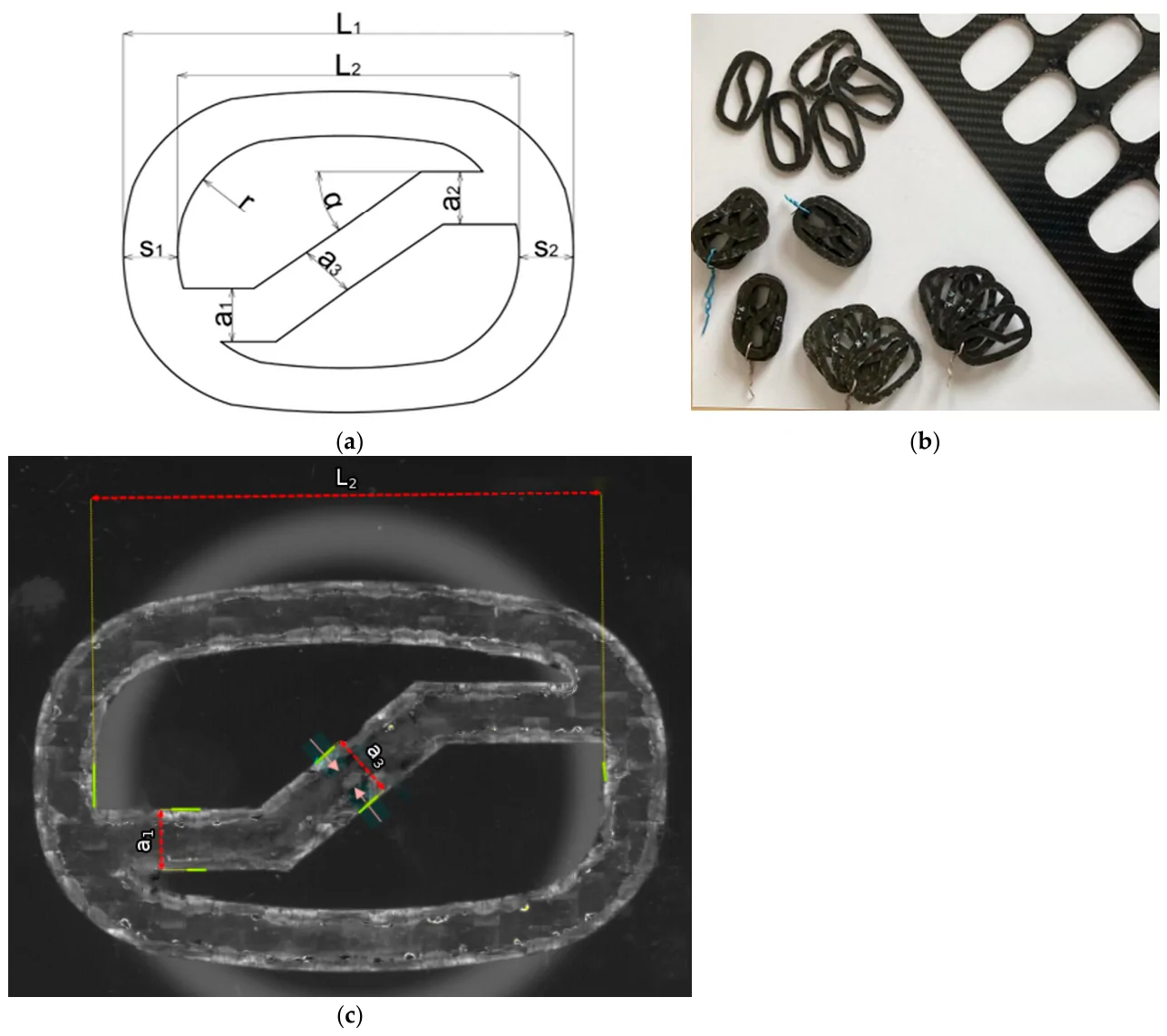
(**a**) CAD geometry of the CFRP test specimen with the evaluated dimensional characteristics; (**b**) representative CFRP specimens produced during the experimental campaign; (**c**) enlarged view of cut specimen with *L*_2_, *a*_1_, and *a*_3_ labelled.

The complete specimen geometry is shown in Figure 2a. A total of nine geometrical characteristics were evaluated: the outer width *L*_1_; inner width *L*_2_; three internal feature heights *a*_1_, *a*_2_, and *a*_3_; two wall widths *s*_1_ and *s*_2_; the internal radius *r*; and the inclination angle *α*. Their nominal values were defined by the CAD model and were identical for all specimens. For the three responses analyzed in this study, the nominal values were *L*_2_ = 31.83 mm, *a*_1_ = 4.00 mm and *a*_3_ = 4.37 mm. Following the predefined experimental matrix, the specimens were produced using the specific combinations of laser power, cutting speed, and operating mode. Representative laser-cut CFRP specimens obtained during the experimental campaign are shown in Figure 2b,c.

### 2.5. Dimensional Measurement Methodology

**Figure 3:**
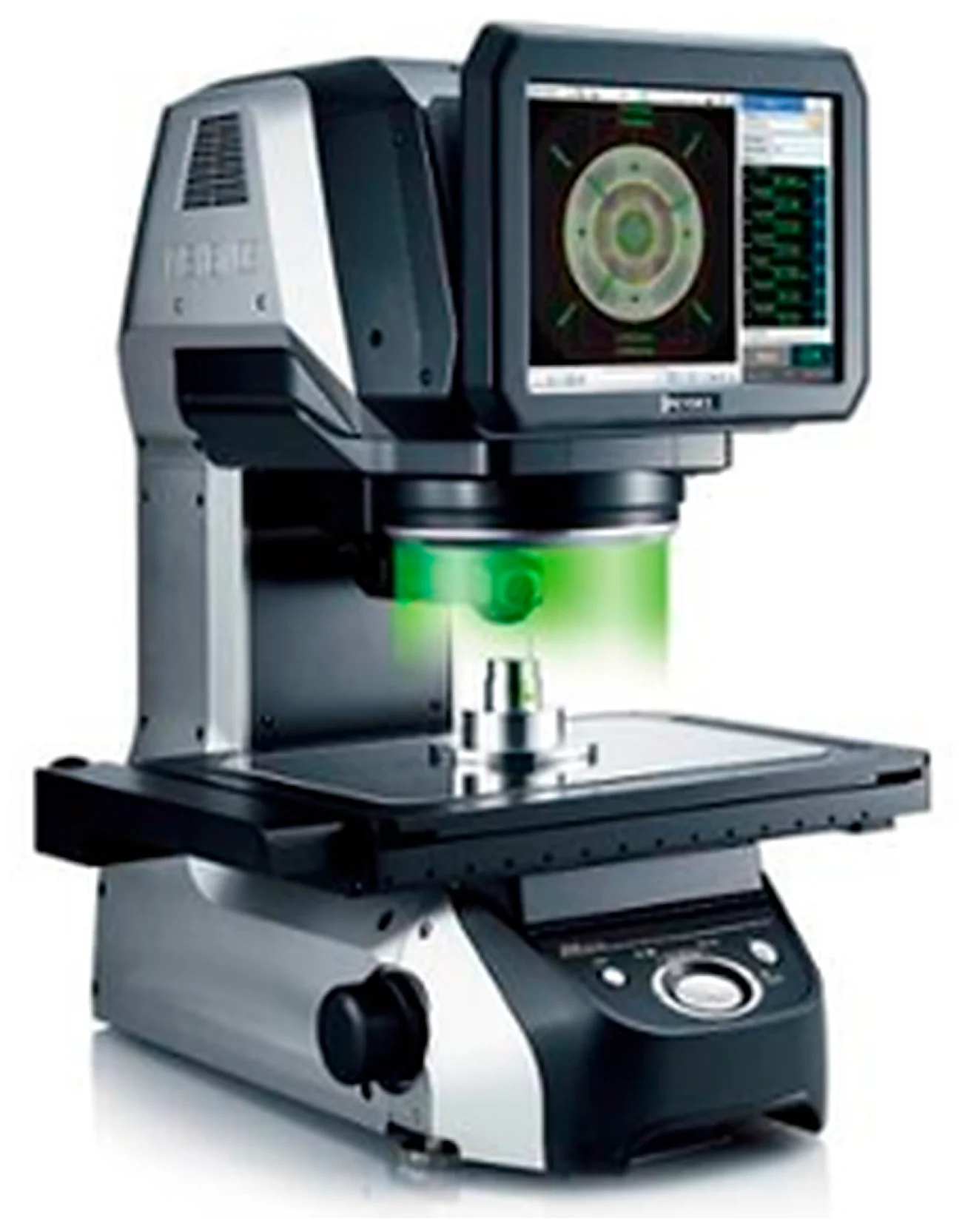
Keyence IM-7500 image dimension measurement system equipped with an IM-7030 measurement head used for dimensional evaluation of the CFRP specimens [18].

Dimensional measurements were performed using a Keyence IM-7500 image dimension measurement system (Keyence, Mouterijpad 11-13, 2800 Mechelen, Belgium) equipped with an IM-7030 measurement head (Figure 3). The system was employed for the automated optical evaluation of the linear, radial, and angular characteristics defined by the specimen geometry.

According to the manufacturer specifications, the IM-7030 measurement head provides a field of view of 300 mm × 200 mm in wide-field mode and 225 mm × 125 mm in the high-precision mode, with a minimum displayed measurement increment of 0.1 µm. Depending on the selected measurement mode, the specified measurement accuracy is up to ±2 µm, with repeatability up to ±0.5 µm [18].

To ensure consistent evaluation of the complete experimental dataset, a dedicated measurement program was prepared prior to data acquisition. The program contained predefined geometrical features corresponding to the nominal CAD geometry and was applied identically to all specimens. This approach minimized operator intervention and ensured consistent feature recognition and measurement throughout the experimental campaign.

Dimensional deviation was calculated as the difference between the nominal CAD dimension and the measured dimension, Δ*x* = *x_nominal_* − *x_measured_*. Accordingly, positive deviation indicates that the measured feature is smaller than its nominal dimension, whereas negative deviation indicates that the measured dimension exceeds the nominal value. 

A total of 90 laser-cut specimens were evaluated. For each specimen, the geometrical characteristics *L*_2_, *a*_1_, and *a*_3_, were recorded using the predefined measurement routine. The resulting measurement data were subsequently exported for statistical processing in Minitab 22.

### 2.6. Statistical Analysis

The measured data were processed using statistical methods to evaluate the significance of individual process parameters and their interaction effects on the selected responses. The analysis included the evaluation of main effects and interaction effects, supported by analysis of variance (ANOVA) to identify statistically significant factors [19].

The measured data were statistically analyzed to determine the influence of the investigated process parameters and their interactions on the selected dimensional responses. Main and interaction effects were evaluated using analysis of variance (ANOVA), enabling the identification of statistically significant factors [19].

All statistical analyses were performed using Minitab 22. Parameter significance was assessed based on p-values and standardized effects, while regression models were developed to quantify the relationship between the process parameters and dimensional deviations. These models were subsequently used to evaluate process trends and support the prediction of dimensional accuracy within the investigated experimental domain.

The mean of the replicates at each of the 18 experimental settings was used as the response in the ANOVA and regression analysis (n = 18). Prior to model development, the distribution and variability of the experimental data were examined using histograms and box plots. ANOVA was subsequently performed to determine the statistical significance of the investigated process factors—cutting speed (*v*), laser power (*P*), and laser operating mode (*M*)—and their interactions. A significance level of *α* = 0.05 was adopted. Main-effects and interaction plots were used to support interpretation of the individual and combined effects of the processing parameters on the resulting dimensional deviations.

Regression models were developed independently for the evaluated geometrical responses. Statistically significant main effects, interaction terms, and, where appropriate, higher-order terms were retained to describe the relationship between the processing conditions and dimensional deviation [19,20]. Model quality and predictive capability were evaluated using the coefficient of determination (*R*^2^), the predicted coefficient of determination ($R_{\mathrm{pred}}^{2}$), and residual diagnostics. Predictive capability was assessed by leave-one-out cross-validation, in which each setting was omitted in turn; the model was refitted to the remaining 17 settings, and the omitted setting was predicted. The experimental analysis included verification of residual normality and independence prior to interpretation of the resulting regression models. 

The complexity of the regression model was selected separately for each dimensional response according to the statistically significant effects observed within the investigated experimental domain. Accordingly, linear, interaction, or quadratic terms were retained only where supported by the experimental data. The resulting equations should therefore be interpreted as empirical models describing the investigated processing window rather than as universal representations of the physical laser–material interaction. 

In addition, the possible influence of experimental run order was evaluated by examining the model residuals as a function of cutting sequence to identify potential systematic temporal trends during the experimental campaign. 

For responses exhibiting statistically meaningful dependence on the quantitative process factors, three-dimensional response-surface plots were generated to visualize the combined influence of laser power and cutting speed within the investigated experimental domain. These plots were used to support interpretation of parameter interactions, nonlinear trends, and regions associated with reduced dimensional deviation [20].

## 3. Results

The experimental data were statistically analyzed to assess the effects of cutting speed, laser power, and laser operating mode on the dimensional accuracy of the CFRP specimens produced by fiber laser cutting. Although multiple geometrical characteristics were measured during the experimental investigation, the detailed analysis presented in this section focuses on the responses *L*_2_, *a*_1_, and *a*_3_. These characteristics provided robust statistical models and represent geometrically different features of the investigated specimen.

The analysis comprised the evaluation of dimensional responses, individual and combined parameter effects, and analysis of variance (ANOVA), Pareto analysis of standardized effects, and regression modeling. Three-dimensional response-surface plots based on the developed regression models were subsequently used to visualize the combined influence of cutting speed and laser power within the investigated experimental domain.

### 3.1. Evaluation of Dimensional Deviations

Table 2 gives the mean deviations of *L*_2_, *a*_1_ and *a*_3_ from their nominal values (31.83 mm, 4.00 mm and 4.37 mm), defined as nominal minus measured value. The inner width *L*_2_ was larger than nominal in all 18 settings (measured: 31.883–31.944 mm; deviations from −0.053 to −0.114 mm), whereas *a*_1_ and *a*_3_ were smaller than nominal (deviations of 0.109–0.180 mm and 0.118–0.171 mm, respectively). This difference indicates that individual geometrical features respond differently to the thermal and material-removal conditions generated during fiber laser cutting. The standard deviation of the five replicates was small (pooled values of 0.0030 mm for *L*_2_, 0.0037 mm for *a*_1_ and 0.0048 mm for *a*_3_) and three to six times smaller than the standard deviation of the setting means (0.016–0.018 mm), indicating that the differences between settings exceeded the replicate scatter.

Distinct parameter-dependent trends were observed among the three responses. For *L*_2_ and *a*_1_, the magnitude of the deviation generally increased with laser power and decreased with cutting speed. For example, at 250 mm/min and in the continuous-wave mode, Δ*L*_2_ changed from −0.082 mm at 800 W to −0.112 mm at 2000 W, and Δ*a*_1_ from 0.142 to 0.180 mm. In contrast, *a*_3_ exhibited a more pronounced nonlinear behavior, particularly with respect to cutting speed: its deviation reached a maximum at 500 mm/min in all six combinations of laser power and operating mode. The smallest deviations of *L*_2_ and *a*_1_ occurred at 1000 mm/min, 800 W, pulsed mode (setting 14), whereas the smallest deviation of *a*_3_ occurred at 250 mm/min, 800 W, continuous-wave mode (setting 1), with setting 14 close behind (0.120 mm). These differences demonstrate that dimensional accuracy therefore cannot be described by a single general relationship for the complete component and support the use of geometry-specific statistical models.

### 3.2. Influence of Process Parameters on Dimensional Accuracy

**Figure 4:**
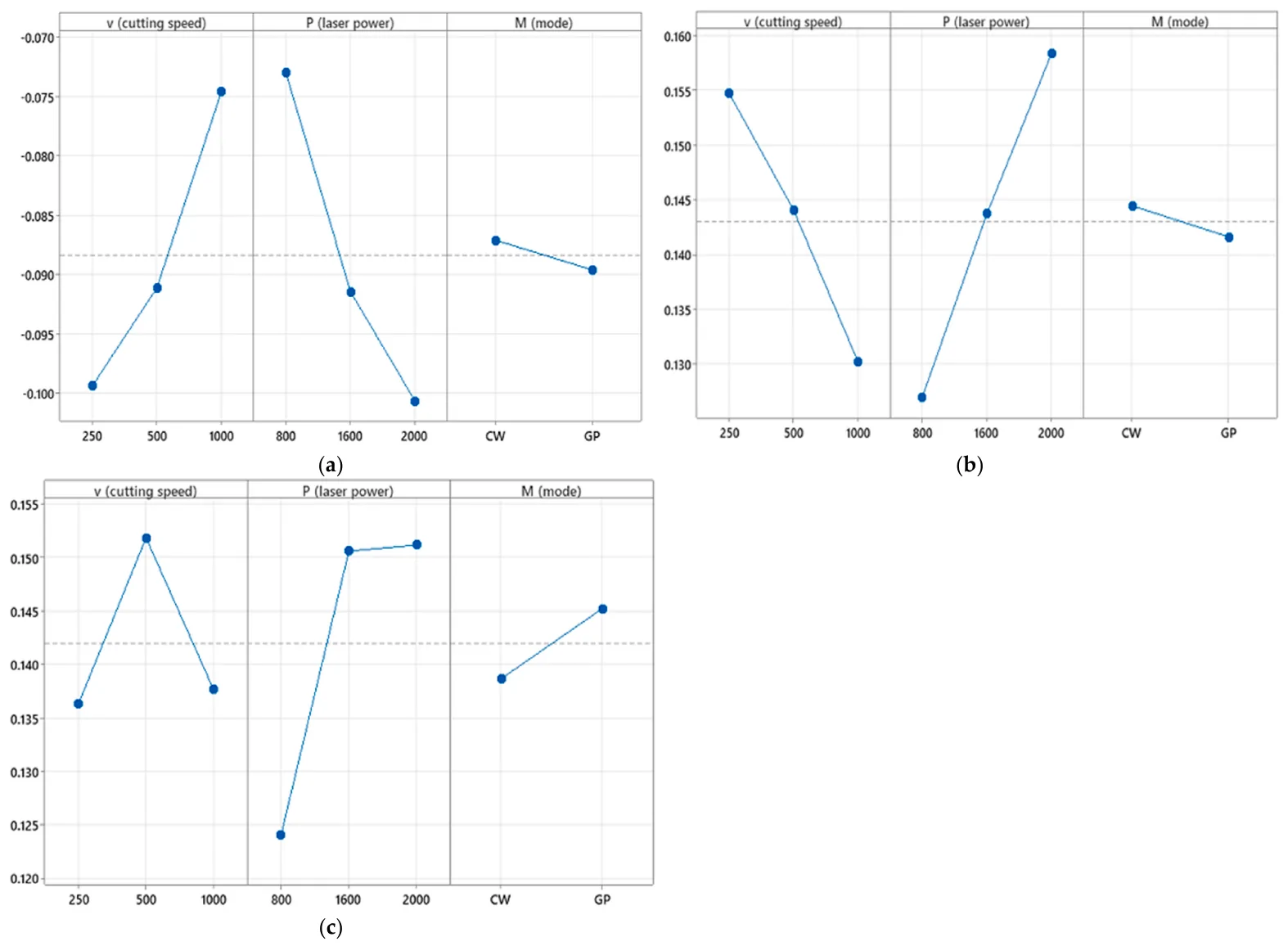
Main effects plots illustrating the influence of cutting speed, laser power, and laser operating mode on dimensional deviation: (**a**) *L*_2_, (**b**) *a*_1_, (**c**) *a*_3_. The horizontal axis represents the investigated levels of cutting speed *v* (mm/min), laser power *P* (W), and operating mode (CW or GP), while the vertical axis represents the corresponding dimensional deviation (mm).

The main-effects plots presented in Figure 4 illustrate the influence of cutting speed (*v*), laser power (*P*), and laser operating mode (*M*) on the three selected dimensional responses. The slope between individual factor levels provides a direct indication of the relative influence of each parameter.

For *L*_2_, laser power produced the strongest main effect. Increasing the laser power from 800 to 2000 W resulted in progressively larger negative dimensional deviations. This trend is consistent with kerf widening at higher energy input, which enlarges the internal opening (Section 4.1 and Section 4.2). Cutting speed showed the opposite trend: increasing the speed from 250 to 1000 mm/min moved the response closer to zero, indicating improved dimensional accuracy. The operating mode produced a smaller but still statistically relevant change, with the GP mode resulting in slightly more negative mean deviations than the CW mode. The small difference between the two regimes is related to the high duty cycle of the pulsed mode (Section 4.4).

For *a*_1_, laser power was again the dominant factor, followed by cutting speed. The mean dimensional deviation increased progressively with laser power, while increasing cutting speed substantially reduced the response. Since *a*_1_ is a solid feature bounded by cut edges, a wider kerf reduces its dimension, which explains why the deviation grows with energy input (Section 4.1 and Section 4.2). The difference between CW and GP operation was comparatively small, suggesting only a limited influence of operating mode on this geometrical feature.

The behavior of *a*_3_ was distinctly different. Cutting speed exhibited a pronounced nonlinear response, with the highest mean deviation occurring at the intermediate speed of 500 mm/min. Laser power also significantly influenced *a*_3_, with a strong increase between 800 and 1600 W followed by only a comparatively small additional change at 2000 W. The GP mode resulted in a higher mean deviation than CW operation. Possible reasons for the maximum at 500 mm/min, namely heat accumulation in the narrow web and the changed cutting direction relative to the fibers, are discussed in Section 4.3.

Figure 4 summarizes these main effects and enables direct comparison of the sensitivity of *L*_2_, *a*_1_, and *a*_3_ to the investigated processing parameters. While *L*_2_ and *a*_1_ exhibit comparatively systematic trends with cutting speed and laser power, the *a*_3_ response demonstrates clear curvature, indicating that a purely linear model would be insufficient for describing this geometrical characteristic.

### 3.3. Influence of Parameter Interactions

**Figure 5:**
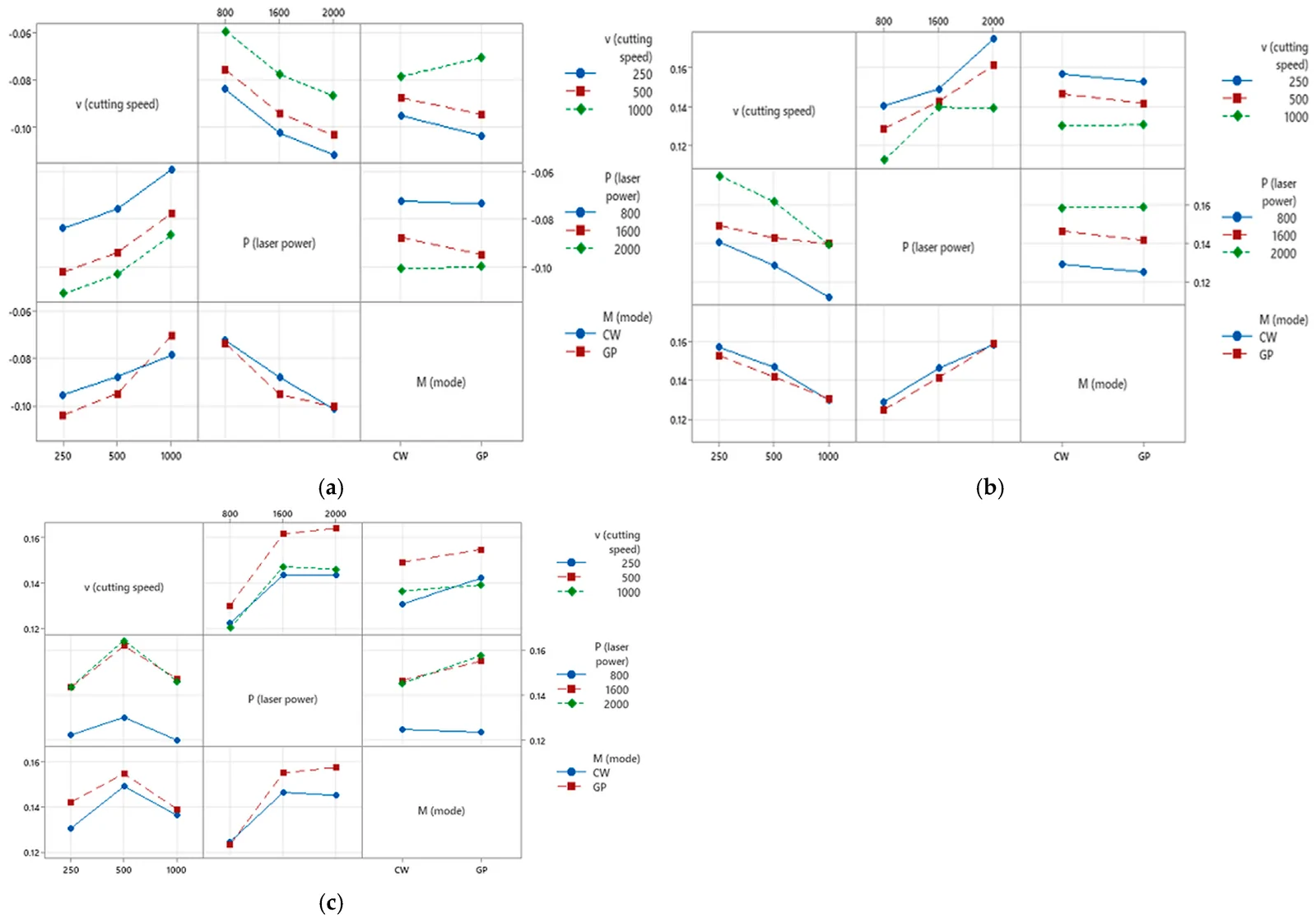
Interaction plots showing the combined effects of cutting speed, laser power, and laser operating mode on dimensional deviation: (**a**) *L*_2_, (**b**) *a*_1_, (**c**) *a*_3_.

The interaction plots shown in Figure 5 were used to evaluate whether the effect of one processing parameter depended on the level of another parameter. The analysis revealed response-dependent interaction behavior, with the most pronounced statistically significant interaction occurring for *L*_2_.

For *L*_2_, a statistically significant interaction was identified between cutting speed and laser operating mode (*v* × *M*), with a p-value of 0.026. This indicates that the influence of cutting speed on dimensional deviation differs between CW and GP operation. The regression coefficients further confirm this behavior, since the slope associated with cutting speed changes according to the selected operating mode. The speed slope was about twice as steep in GP as in CW operation; so, the improvement with increasing speed is stronger in the pulsed regime. Because the overall CW–GP difference is small, this interaction should be confirmed at lower duty cycles (Section 4.4).

For *a*_1_, the interaction plot shows comparatively similar trends between factor combinations. No interaction term was retained in the final regression model, indicating that the response can be adequately described primarily through the independent effects of cutting speed and laser power. The effect of operating mode was also statistically insignificant (p = 0.407). This agrees with the near-identical thermal input of CW and GP operation at a 99% duty cycle (Section 4.4).

The *a*_3_ response exhibited more complex process behavior. However, the final model did not require cross-interaction terms between the investigated factors. Instead, the curvature observed in the experimental response was adequately represented by the quadratic terms of cutting speed and laser power. This suggests that the complexity of the *a*_3_ response is associated primarily with nonlinear parameter effects rather than with strong two-factor interactions. The curvature is discussed in Section 4.3.

### 3.4. Statistical Analysis of Process Parameters

**Figure 6:**
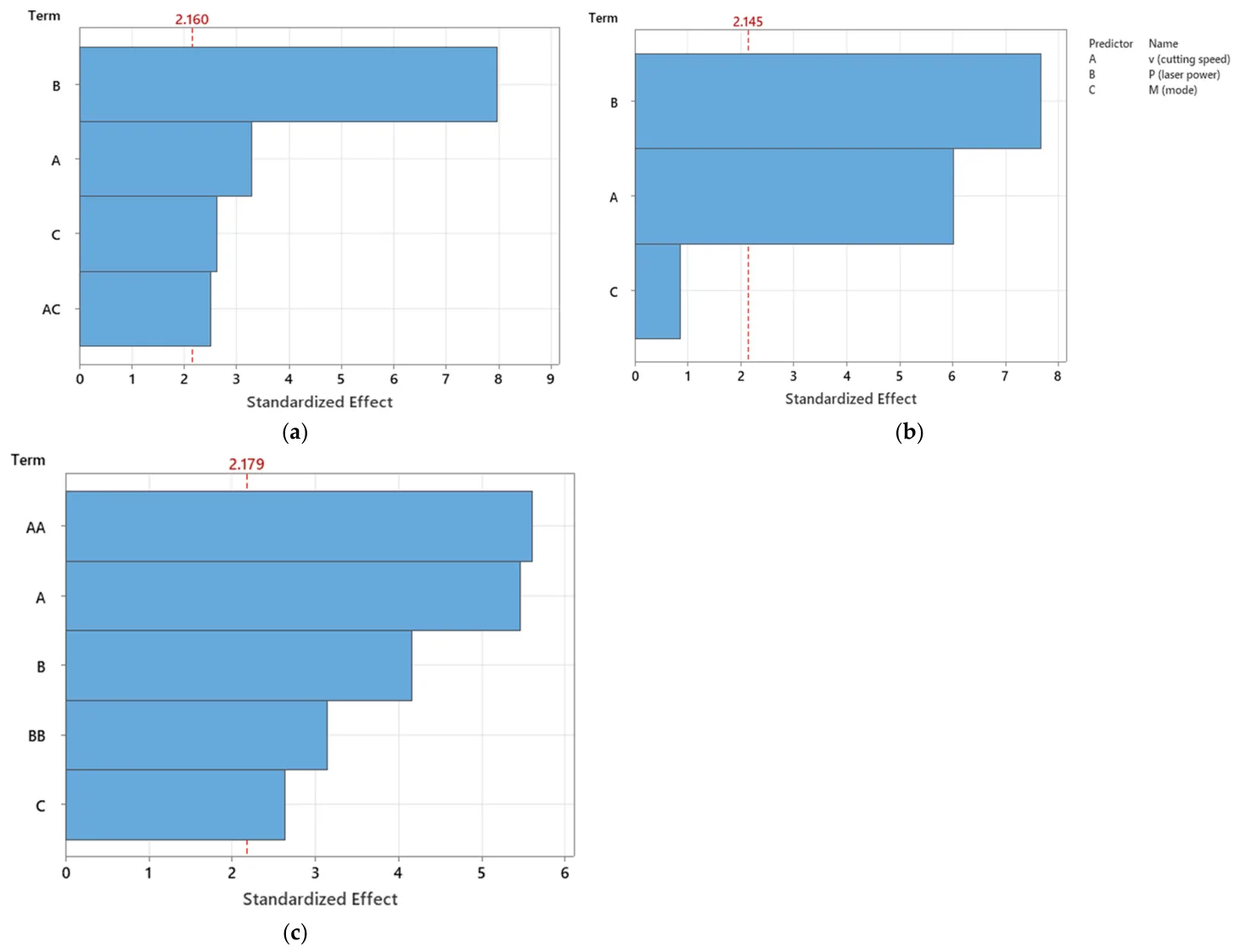
Pareto charts showing the relative importance and statistical significance of the investigated process parameters and model terms for: (**a**) *L*_2_, (**b**) *a*_1_, (**c**) *a*_3_.

The significance of investigated process parameters was statistically assessed by analysis of variance (ANOVA) at the significance level of α = 0.05. The standardized effects of the individual model terms are illustrated by the Pareto charts in Figure 6, while the principal ANOVA results and model-performance indicators are summarized in Table 3.

For *L*_2_, laser power was identified as the dominant factor (F = 63.73, p < 0.001), followed by cutting speed (F = 10.80, p = 0.006). The laser operating mode was also statistically significant (p = 0.021). These findings show that the effect of cutting speed on *L*_2_ depends partly on the selected laser operating regime.

For *a*_1_, laser power again exhibited the strongest effect (F = 58.82, p < 0.001), while cutting speed was also highly significant (F = 36.29, p < 0.001). In contrast, the laser operating mode was statistically insignificant (p = 0.407), confirming that the dimensional response of *a*_1_ is governed predominantly by laser power and cutting speed.

The *a*_3_ response exhibited a more complex nonlinear behavior. Cutting speed (p < 0.001), laser power (p = 0.001), and operating mode (p = 0.022) were statistically significant. In addition, both quadratic terms contributed significantly to the model, with *v*^2^ showing the strongest effect (F = 31.47, p < 0.001) and *P*^2^ also remaining significant (F = 9.90, p = 0.008). The significance of the quadratic terms confirms the nonlinear dependence observed in the main-effects analysis.

The coefficients of determination demonstrate good agreement between the experimental data and the developed regression models. The highest *R*^2^ was obtained for *a*_3_ (92.22%), followed by *L*_2_ (90.37%) and *a*_1_ (87.25%). The relatively small differences between *R*^2^, adjusted *R*^2^, and predicted *R*^2^ indicate that the models retain satisfactory predictive capability within the investigated experimental domain.

Leave-one-out cross validation further confirmed the predictive accuracy of the developed models. The cross-validated mean absolute errors (MAE) were 0.00684, 0.00644, and 0.00481 mm for *L*_2_, *a*_1_, and *a*_3_, respectively, while the corresponding root mean square errors (RMSE) were 0.00713, 0.00794, and 0.00646 mm. These errors are small relative to the observed dimensional-deviation ranges and support the predictive applicability of the models within the investigated experimental domain. 

The residuals were also plotted as a function of the order of the experimental runs to see if a systematic sequence-dependent bias had been introduced by gradual changes in the cutting system. No progressive trend or systematic change in the residuals was observed along the experimental sequence. This suggested that the order of cutting and possible time-dependent modifications of the optical system had no detectable effect on the computed dimensional answers.

### 3.5. Regression Analysis and Response Surfaces

Regression analysis was conducted to characterize the relationship between the investigated process parameters and the selected dimensional responses. Since laser operating mode is a categorical factor, separate equations are presented for continuous-wave (CW) and gated-pulsed (GP) operation where applicable.

For graphical interpretation, the three-dimensional fitted regression surfaces are presented only for the CW mode. This mode was selected as a representative condition to provide a clear visualization of the combined effects of the two continuous process parameters, laser power and cutting speed, without duplicating analogous response-surface plots for both operating modes. The effect of the GP mode is retained in the statistical analysis and is fully described by the corresponding mode-specific regression equations. Accordingly, the graphical representation is intended to highlight the relationship between the continuous process variables and the dimensional responses while preserving the effect of operating mode within the statistical models.

The required model complexity differed between the evaluated responses. Although nonlinear and synergistic phenomena are inherent to laser processing of heterogeneous CFRP materials, the statistical models were developed to reproduce the response observed within the investigated parameter domain. For *L*_2_, a significant interaction between cutting speed and operating mode was retained, whereas the *a*_1_ response was adequately represented by the independent effects of cutting speed and laser power. In contrast, the pronounced curvature observed for *a*_3_ required the inclusion of quadratic terms. Thus, the linear form of the *L*_2_ and *a*_1_ equations should not be interpreted as evidence of globally linear laser–material interaction, but rather as a local empirical representation of these dimensional responses over the investigated processing range. 

For the *L*_2_ response, the regression equations are:

*L*_2, CW_ = −0.06590 + 0.00002143*v* − 0.00002300*P* (1)

and

*L*_2, GP_ = −0.08190 + 0.00004457*v* − 0.00002300*P* (2)

The negative coefficient of laser power indicates that increasing *P* shifts the *L*_2_ response toward larger negative dimensional deviations. In contrast, increasing cutting speed shifts the response toward less negative values. The different cutting-speed coefficients obtained for CW and GP operation reflect the statistically significant interaction between cutting speed and operating mode identified by the ANOVA. Therefore, although the dependence on the continuous variables is approximately linear within the investigated range, the *L*_2_ model still captures a synergistic effect through the significant *v* × *M* interaction. The model achieved *R*^2^ = 90.37%, $R_{\mathrm{adj}}^{2}$ = 87.41% and $R_{\mathrm{pred}}^{2}$ = 81.79%.

**Figure 7:**
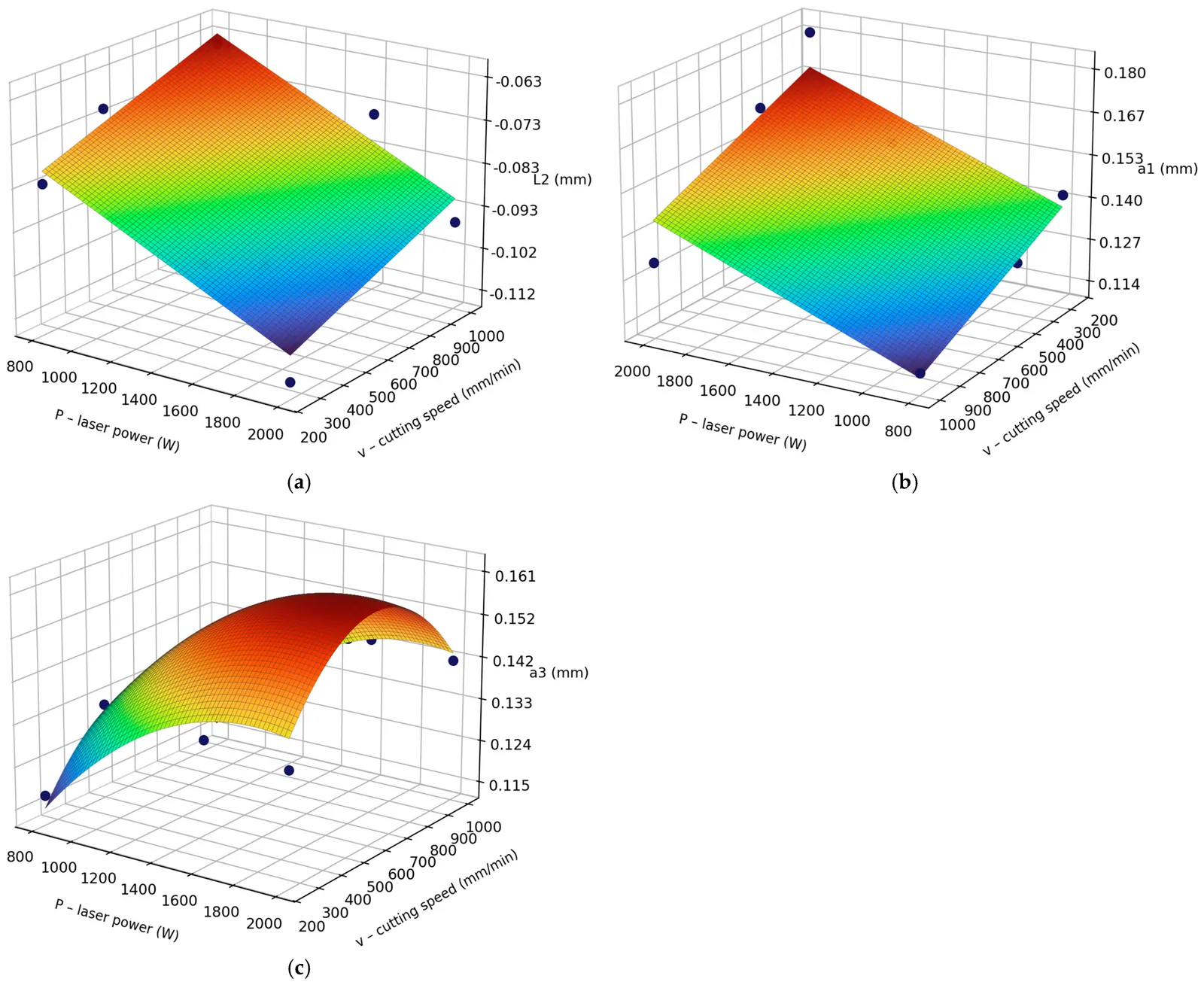
Fitted three-dimensional regression surfaces illustrating the combined influence of laser power P and cutting speed *v* on the selected dimensional responses under CW operation: (**a**) *L*_2_, (**b**) *a*_1_, (**c**) *a*_3_. The surfaces represent the developed regression models, while the experimental mean responses are indicated by individual points. Regression equations for both CW and GP modes are provided in Equations (1)–(6).

The fitted regression surface for CW operation is shown in Figure 7a. Within the investigated parameter range, the response exhibits an approximately planar behavior. Higher laser power combined with lower cutting speed produces larger negative deviations, whereas lower power and higher cutting speed shift the predicted response toward zero.

For *a*_1_, the regression equations are:

*a*_1, CW_ = 0.12582 − 0.00003191*v* + 0.00002539*P* (3)

and

*a*_1, GP_ = 0.12300 − 0.00003191*v* + 0.00002539*P* (4)

The regression coefficients demonstrate opposing effects of the continuous process parameters. Increasing cutting speed reduces the dimensional deviation, whereas increasing laser power increases it. Only a small difference was observed between the CW and GP intercepts, which is consistent with the statistically insignificant influence of operating mode on *a*_1_. The model achieved *R*^2^ = 87.25%, $R_{\mathrm{adj}}^{2}$ = 84.52% and $R_{\mathrm{pred}}^{2}$ = 78.95%.

For *a*_1_, the approximately monotonic experimental response and the satisfactory predictive performance of the reduced model indicate that additional model complexity was not required to describe the response within the investigated parameter range. This does not exclude nonlinear behavior outside the studied processing window. 

As illustrated in Figure 7b, the fitted response is nearly planar. The lowest predicted *a*_1_ values occur at lower laser power and higher cutting speed, whereas the opposite parameter combination leads to increased dimensional deviation.

In contrast to *L*_2_ and *a*_1_, the *a*_3_ response exhibited a pronounced nonlinear dependence on the investigated process parameters. Therefore, quadratic terms were included in the regression model.

The resulting equations are:

*a*_3, CW_ = 0.0244 + 0.0001523*v* + 0.0000965*P* − 1.204 × 10^−7^*v*^2^ − 2.639 × 10^−8^*P*^2^ (5)

and

*a*_3, GP_ = 0.0309 + 0.0001523*v* + 0.0000965*P* − 1.204 × 10^−7^*v*^2^ − 2.639 × 10^−8^*P*^2^ (6)

The significant quadratic terms result in the curved fitted regression surface presented in Figure 7c. The *a*_3_ model exhibited the highest goodness of fit among the evaluated responses, with *R*^2^ = 92.22%, $R_{\mathrm{adj}}^{2}$ = 88.98% and $R_{\mathrm{pred}}^{2}$ = 82.49%.

Unlike the approximately planar surfaces obtained for *L*_2_ and *a*_1_, the *a*_3_ response reaches higher predicted values within an intermediate cutting-speed region and at relatively high laser power. The subsequent decrease toward higher cutting speeds is consistent with the statistically significant quadratic contribution of cutting speed identified in the ANOVA.

Overall, the regression analysis demonstrates that the dimensional response depends strongly on the evaluated geometrical characteristic. The responses *L*_2_ and *a*_1_ are governed predominantly by linear effects of laser power and cutting speed, whereas *a*_3_ exhibits a distinctly nonlinear behavior. Although only the CW response surfaces are presented graphically for clarity and to avoid redundant visualization, the influence of both operating modes is incorporated in the developed regression models and statistical evaluation.

### 3.6. Optical Examination of the Cut-Edge Morphology 

**Figure 8:**
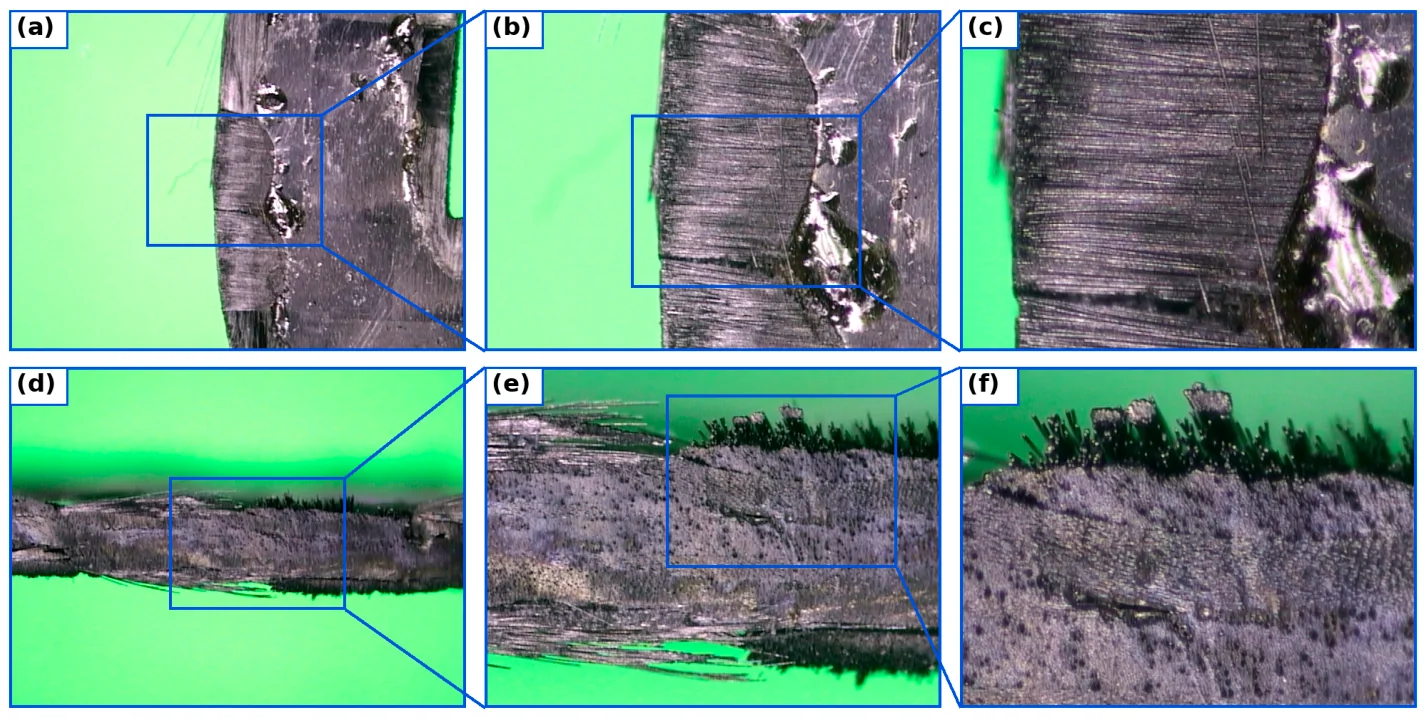
Optical micrographs of representative laser-cut CFRP edges viewed (**a**–**c**) along the laser-beam direction and (**d**–**f**) perpendicular to the laser-beam direction at increasing magnifications: (**a**,**d**) 33.9×, (**b**,**e**) 88.5×, and (**c**,**f**) 176×. The images illustrate local edge irregularities, adhered or redeposited material, and exposed or protruding carbon fibers associated with non-uniform material removal.

Representative optical micrographs of the laser-cut CFRP edges are presented in Figure 8. Images acquired along the laser-beam direction revealed a locally irregular cut boundary and regions of adhered or redeposited material adjacent to the cut edge. The higher-magnification observations showed that the material-removal boundary was not completely uniform along the contour.

Micrographs obtained perpendicularly to the laser-beam direction revealed a heterogeneous cut-edge morphology through the laminate thickness. Protruding and locally exposed carbon fibers were observed along the cut boundary, together with regions in which the surrounding matrix appeared to have been removed to a greater extent than the fibers. These observations are consistent with preferential thermal degradation and recession of the polymer matrix relative to the carbon-fiber reinforcement. The resulting non-uniform edge morphology provides qualitative support for the geometry-dependent dimensional deviations observed in the optical measurements.

Because the present observations were obtained by optical microscopy rather than SEM, they should be interpreted as qualitative morphological evidence and not as direct microstructural confirmation of the proposed degradation mechanisms. 

## 4. Discussion

The results show that the dimensional accuracy achieved during fiber laser cutting of CFRP is influenced by both the selected process parameters and the specific geometrical feature being evaluated.

### 4.1. Kerf Formation and the Sign of the Dimensional Deviations

The opposite signs of the dimensional deviations can primarily be explained by the geometrical relationship between the programmed contour and the material removed by the laser beam. Since no kerf compensation was applied, the laser beam followed the nominal CAD contour. Material removal on both sides of the programmed path therefore enlarges internal openings while reducing the dimensions of solid features bounded by cut edges. According to the adopted deviation definition, this produces negative deviations for the inner width *L*_2_ and positive deviations for the solid features *a*_1_ and *a*_3_. 

The magnitude of these deviations is additionally influenced by the heterogeneous thermal response of CFRP. The polymer matrix undergoes thermal degradation at substantially lower temperatures than the carbon fibers, and preferential matrix removal may extend beyond the region of direct fiber ablation. This matrix recession effectively shifts the detected cut edge and increases the apparent material-removal width. The optical micrographs presented in Figure 8 provide qualitative support for this interpretation. In particular, the cross-sectional views perpendicular to the laser-beam direction show locally exposed and protruding fiber ends together with non-uniform recession of the surrounding material. Such morphology is consistent with preferential thermal degradation of the polymer matrix and an unequal removal response of the matrix and carbon fibers. At the same time, the strongly anisotropic thermal conductivity of the carbon-fiber reinforcement causes heat to propagate preferentially along the fiber direction, making the local edge response dependent on the orientation of the cutting path relative to the reinforcement [9,10]. Consequently, the sign of the deviation is primarily determined by feature geometry, whereas its magnitude depends on the local kerf formation, matrix recession, and anisotropic heat-transfer conditions. 

### 4.2. Effect of Energy Input 

Increasing the laser power from 800 to 2000 W increased the mean magnitude of the deviation of *L*_2_ from 0.073 to 0.101 mm and of *a*_1_ from 0.127 to 0.158 mm, while increasing the cutting speed from 250 to 1000 mm/min reduced the deviation of *L*_2_ from 0.099 to 0.075 mm and of *a*_1_ from 0.155 to 0.130 mm. Since the deviations reflect the kerf width, these trends indicate that the kerf widened with increasing energy input per unit length, expressed as the nominal line energy (E_L)_ (Table 4):

(7) $$E_{L}\;=\;\frac{P}{v}$$

where *P* is the laser power in watts and *v* is the cutting speed in mm/s.

The line energy varied tenfold (48–480 J/mm), and its highest values were associated with the largest deviations of *L*_2_ and *a*_1_, consistent with the increasing thermal deformation of CFRP at higher power and lower speed reported by Ma et al. [21]. Across the 18 settings, the correlation between line energy and the deviation magnitude was 0.82 for *L*_2_ and 0.84 for *a*_1_, but only 0.27 for *a*_3_, which indicates that line energy alone does not explain the *a*_3_ response (Section 4.3). 

Higher power and longer interaction time at lower speed extend this region, and the optical system records the receded edge as a dimensional deviation. 

The replicate scatter was small for all three responses (median within-setting standard deviation of 0.003 mm for *L*_2_, 0.004 mm for *a*_1_ and 0.004 mm for *a*_3_), roughly an order of magnitude smaller than the effects of laser power (0.028 mm for *L*_2_ and 0.031 mm for *a*_1_) and cutting speed (0.025 mm and 0.024 mm, respectively). The estimated variation in the focal and stand-off positions of approximately ±0.2 mm caused by the limited flatness of the laminates (Section 2.2) therefore did not produce large scatter in the measured dimensions.

### 4.3. Nonlinear Response of a_*3*_

The deviation of *a*_3_ reached a maximum at the intermediate speed of 500 mm/min (0.152 mm), compared with 0.136 and 0.138 mm at 250 and 1000 mm/min, respectively. It also increased with laser power from 0.125 mm at 800 W to 0.151 mm at 1600 W, with almost no further change at 2000 W (0.151 mm). Since line energy decreases monotonically with speed (Table 4), this behavior indicates competing mechanisms. Two features of the *a*_3_ geometry may contribute to this: heat accumulation in the narrow diagonal web, which is cut on both sides, and the inclination of the web (α = 38.66°), which changes the cutting direction relative to the fibers and thus the anisotropic heat conduction [2,8]. Confirming the dominant mechanism requires local measurements of kerf width and heat-affected zone along the web.

### 4.4. Effect of the Operating Mode 

The effect of the operating mode was statistically significant for *L*_2_ and *a*_3_ but not for *a*_1_, and small in absolute terms: the mean difference between GP and CW was 0.003 mm for *L*_2_, 0.003 mm for *a*_1_ and 0.007 mm for *a*_3_ (GP gave larger deviations for *L*_2_ and *a*_3_ and a slightly smaller one for *a*_1_). For *L*_2_, the speed slope differed between the two modes, being about twice as steep in GP as in CW operation, which corresponds to the significant speed × mode interaction (Section 3.3). The small overall CW–GP differences are attributed to the high duty cycle (99%) in GP operation. At 5000 Hz, the off-time was only about 2 µs per period; so, the thermal input was nearly identical to CW operation. Larger differences can be expected at lower duty cycles, which allow partial cooling between pulses.

### 4.5. Practical Implications 

Unlike previous studies focused on kerf width, heat-affected zone, and edge integrity [2,8,22], the present results show how laser power, cutting speed, and operating mode translate into deviations of individual component features. Lower laser power and higher cutting speed, corresponding to lower line energy, reduced the deviations of *L*_2_ and *a*_1_, whereas *a*_3_ was the lowest at the lowest and highest cutting speeds. As no single parameter combination minimized all responses simultaneously, process parameters should be selected with respect to the critical feature of the component. 

## 5. Conclusions

This study demonstrated that laser power, cutting speed, and operating mode affect the dimensional accuracy of fiber-laser-cut CFRP in a geometry-dependent manner. For *L*_2_ and *a*_1_, laser power was the dominant factor, followed by cutting speed, with higher power increasing and higher speed reducing the dimensional deviation. In contrast, *a*_3_ exhibited a pronounced nonlinear dependence on cutting speed, with the highest deviation occurring at 500 mm/min. The operating mode had a statistically significant but comparatively small effect on *L*_2_ and *a*_3_, while no significant effect was observed for *a*_1_. 

Among the investigated conditions, 800 W and 1000 mm/min in the GP mode provided the most favorable overall dimensional performance, producing the lowest deviations of *L*_2_ (−0.0533 mm) and *a*_1_ (0.1086 mm) and a near-minimum deviation of *a*_3_ (0.120 mm). The minimum *a*_3_ deviation of 0.118 mm was obtained at 800 W and 250 mm/min in the CW mode, confirming that the optimum processing condition depends on the geometrical feature considered. The developed regression models showed good agreement with the experimental data (*R*^2^ = 87.25–92.22%), while leave-one-out cross-validation yielded predicted *R*^2^ values of 78.95–82.49% and RMSE values of approximately 0.006–0.008 mm. 

From an engineering perspective, lower laser power combined with higher cutting speed can be recommended as a practical starting condition when *L*_2_ and *a*_1_ accuracy is prioritized, whereas *a*_3_ requires feature-specific optimization. The results also indicate that parameter-dependent kerf compensation could further improve dimensional accuracy. These conclusions are limited to the investigated 1 mm CFRP laminate and processing window; future work should include independent validation, additional laminate configurations, direct kerf and HAZ measurements, and SEM characterization of the cut edges. 

## Figures and Tables

**Table 1:** Investigated process parameters and their experimental levels.

Factor	Symbol	Level 1	Level 2	Level 3
Cutting speed	*v* (mm/min)	250	500	1000
Laser power	*P* (W)	800	1600	2000
Laser operating mode	*M* (-)	CW	GP	-

**Table 2:** Mean dimensional deviations of *L*_2_, *a*_1_ and *a*_3_ for the 18 experimental settings (mean of five replicates). Deviation = nominal − measured value.

Setting No.	Cutting Speed (mm/min)	Laser Power (W)	Mode	Δ*L*_2_ (mm)	Δ*a*_1_ (mm)	Δ*a*_3_ (mm)
1	250	800	CW	−0.0818	0.1418	0.118
2	250	800	GP	−0.0863	0.139	0.1265
3	250	1600	CW	−0.0912	0.149	0.138
4	250	1600	GP	−0.1137	0.149	0.1485
5	250	2000	CW	−0.1124	0.1796	0.136
6	250	2000	GP	−0.1109	0.17	0.151
7	500	800	CW	−0.0703	0.1296	0.132
8	500	800	GP	−0.0813	0.1274	0.128
9	500	1600	CW	−0.0977	0.1472	0.158
10	500	1600	GP	−0.0907	0.138	0.165
11	500	2000	CW	−0.0949	0.163	0.157
12	500	2000	GP	−0.1119	0.1594	0.171
13	1000	800	CW	−0.0653	0.1156	0.124
14	1000	800	GP	−0.0533	0.1086	0.120
15	1000	1600	CW	−0.0747	0.1422	0.143
16	1000	1600	GP	−0.0807	0.137	0.151
17	1000	2000	CW	−0.0959	0.132	0.142
18	1000	2000	GP	−0.0779	0.1462	0.15

**Table 3:** Summary of statistical significance and regression model performance.

Response	Significant Model Terms, p-Value	Non-Significant Term	Most Influential Term(s) (F-Value)	*R* ^2^	$R_{\mathrm{adj}}^{2}$	$R_{\mathrm{pred}}^{2}$
*L*_2_	*P* (p < 0.001), *v* (p = 0.006), *M* (p = 0.021), *v* × *M* (p = 0.026)	-	*P* (63.73)	90.37%	87.41%	81.79%
*a*_1_	*P* (p < 0.001), *v* (p < 0.001)	*M* = 0.407	*P* (58.82)	87.25%	84.52%	78.95%
*a*_3_	*v* (p < 0.001), *P* (p < 0.001), *M* (p = 0.022), *v^2^* (p < 0.001), *P*^2^ (p = 0.008)	-	*v*^2^ (31.47), *v* (29.83)	92.22%	88.98%	82.49%

**Table 4:** Nominal line energy for the investigated combinations of laser power and cutting speed.

Cutting Speed (mm/min)	Cutting Speed (mm/s)	Laser Power (W)	Nominal Line Energy (J/mm)
250	4.17	800	192
		1600	384
		2000	480
500	8.33	800	96
		1600	192
		2000	240
1000	16.67	800	48
		1600	96
		2000	120

## Data Availability

The raw data supporting the conclusions of this article will be made available by the authors on request.
